# Cannabinoid Receptor Activation in the Nucleus Tractus Solitaries Produces Baroreflex-Like Responses in the Rat

**Published:** 2008-09

**Authors:** Murat S. Durakoglugil, Hakan S. Orer

**Affiliations:** *Department of Pharmacology, Faculty of Medicine, Hacettepe University, 06100 Ankara, Turkey*

**Keywords:** cannabinoids, arterial pressure, cardiovascular integration, glutamate, brain stem

## Abstract

The effects of cannabinoids on the baroreflex have been investigated in the nucleus tractus solitarii (NTS). In urethane-anesthetized rats, microinjection of the cannabinoid (CB) receptor agonist WIN 55212-2 (100 mM) into the NTS produced a short lasting decrease in arterial pressure (from 95.2 ± 2.9 to 76.2 ± 1.5, n=5, *P*<0.05) but no change in the heart rate. Another cannabinoid agonist, CP 55940 (100 mM) also caused hypotensive responses (from 90.2 ± 11.3 to 66.4 ± 12.3 mmHg, n=5, *P*<0.05). Simultaneous sympathetic nerve discharge recordings showed suppression prior to the arterial pressure lowering effect of these agonists. Microinjection of the cannabinoid receptor antagonist, AM 281 (70 mM) did not cause any significant change in arterial pressure (from 100.8 ± 12 mmHg to 108.1 ± 12.8 mmHg, n=5, *P*>0.05) though it inhibited the agonist-induced responses. The non-NMDA receptor antagonist, DNQX (4 mM) microinjections antagonized the actions of CB agonist WIN 55212-2. Furthermore, sinoaortic denervation attenuated the responses to CB agonists suggesting an intact baroreflex arc is necessary to elicit CB-mediated effects. Neither WIN 55212-2 nor AM 281, altered baroreceptor reflex activation by bolus phenylephrine (25 microg//kg) injections. These data suggest that cannabinoid receptors in the NTS are not involved in the tonic regulation of the arterial pressure but may have a modulatory role in the baroreceptor reflex integration.

## INTRODUCTION

Marihuana has a long history of consumption for recreational and medical purposes. However, its receptors and endogenous ligands known as endocannabinoids (CBs) are relatively new. CB1 receptor subtype is distributed throughout the central and peripheral nervous system and implicated in many physiological functions.

Cannabinoids are known to produce cardiovascular effects. In humans, acute administration of the primary active constituent of the hemp plant *Cannabis sativa*, delta-9-tetrahydrocannabinol (THC) causes tachycardia without any change in BP whereas its long term use causes supine hypotension and bradycardia (1-3).

Following animal studies, there is a general agreement on the peripheral cannabinoid action, which is the inhibition of noradrenalin release from postganglionic sympathetic neurons. Nevertheless, the effects on the brain stem cardiovascular centers are more complex. Early studies, based on the administration of THC into either the cerebral circulation of dogs or the lateral cerebral ventricle of cats, suggested that the central hypotensive and bradycardic effects were due to the sympathoinhibition (4, 5). The hypotensive effect of a synthetic cannabinoid, delta 6a, 10a dimethyl heptyl tetrahydrocannabinol (DMHP, EA 1476), could be prevented by sectioning of the spinal cord at the cervical level. Moreover, administration of low doses of DMHP to dogs blocked the pressor responses to the occlusion of carotid artery but not to adrenaline (6). In a more recent study intracisternal application of CB agonist WIN 55212-2 revealed two effects on brain stem cardiovascular centers in conscious rabbits: sympathoexcitation and activation of cardiac vagal fibers (7, 8). In the same study, high doses of systemically administered WIN 55212-2, a CB1 agonist, produced central sympathoinhibition.

In order to reveal the centers, which may be, involved in this central sympathoinhibition two putative sites namely the nucleus tractus solitarii (NTS) and rostroventrolateral medulla (rostral VLM) were investigated in a few studies. Commissural and caudal parts of the NTS, where the second order neurons of the baroreceptor reflex are located, shows considerably denser binding for cannabinoid ligands (9). Single-unit activity recorded extracellularly from rat brain slices revealed that subpostremal NTS neurons are THC sensitive (10, 11) and that most of them showed opposite responses to 5HT_3_ receptor agonist 1-phenylbiguanide. Both serotonergic and cannabinoid receptors in the NTS are thought to play a role in nausea and vomiting. Furthermore, serotonergic agonists are also known to elucidate cardiovascular effects when microinjected into the NTS (12, 13). In line with this, Seagard *et al* suggested that endocannabinoids might modulate the duration of the baroreflex and induce sympathoinhibition through presynaptic modulation of GABA release (14). Nevertheless, microinjection of cannabinoids into the NTS did not cause any changes while injection into the rostral ventrolateral medulla (rostral VLM), where sympathoexcitatory projection neurons are located, elicited a small sympathoinhibition (15). In contrast, in another study, microinjection of cannabinoids into the rostral VLM caused sympathoexcitation, an increase in arterial pressure and abolished phrenic nerve activity (16). These conflicting results as to whether cannabinoids do exert an influence in the baroreceptor reflex responses in the NTS has prompted us to perform microinjections of CB-related agents into the NTS at the level of obex, the site where the previous studies have shown to affect baroreceptor reflex (12, 13).

## MATERIALS AND METHODS

### Chemicals

WIN 55212-2, CP 55940 and AM 251 were obtained from Tocris (Bristol, UK), DNQX from Sigma (USA). All drugs were dissolved in dimethylsulfoxide (DMSO).

### General Procedures

Experiments were performed in 33 male Sprague-Dawley rats weighing 250-382 g. Animals were housed in a temperature-controlled room with 12 hrs dark/light cycle. Food and tap water were provided ad libitum, until the night before the surgery when food was removed. All experiments were carried out in accordance with the guidelines on animal use in neuroscience research published by the Society for Neuroscience (USA) and approved by the Hacettepe University Laboratory Animals Ethics Committee (DHEK 2001/1-4).

Rats were anesthetized with urethane (1.1-1.4 mg/kg, i.p.) and a polyethylene catheter was inserted into the right carotid or femoral artery to monitor the blood pressure. Arterial pressure was recorded using a pressure transducer (Transbridge World Precision Instruments, Sarasota, FL) and data were sent to a computer via MacLab 4/s data acquisition unit (AD Instruments, Castle Hill, Australia). Heart rate and mean arterial pressure (MAP) were monitored on-line from the arterial pressure signal using the computer software (Chart v. 3.6, Castle Hill, Australia). The built-in module of the software calculated the MAP as the sum of 1/3 of systolic pressure and 2/3 of diastolic pressure. All data were calculated from the same animal before and after drug application. Bilateral jugular or femoral veins were catheterized for drug administration and for dextran (Rheomacrodex, Baxter-Eczacibasi, Istanbul, Turkey) infusion, which maintained the stability of the blood pressure. Animals were tracheotomized and artificially ventilated (Harvard Apparatus Rodent Respirator, Millis, MA) with room air enriched with O_2_ (100 ml/100 g/min, near 60 strokes/min). End tidal CO_2_ was monitored using a capnometer (Capstar 100, CWE, Ardmore, PA) and maintained between 4 to 5 % of expired gases. Animals were immobilized using gallamine triethiodide (induction: 4 mg/kg, maintenance: 0.4 mg/kg as needed; Sigma, St. Louis, MO). Body temperature was measured with a rectal thermometer and maintained at 38 ± 1°C with a heating lamp.

### Sympathetic nerve discharge (SND) recordings

The left greater splanchnic nerve was isolated retroperitoneally and was tied and cut. Then, the central end was placed onto a bipolar platinum hook electrode bathed in paraffin oil where nerve potentials were recorded using a differential amplifier (Grass Instruments, Quincy, MA). Nerve signal was 0.1-1000 Hz band-pass filtered and sent to an analogue-to-digital converter (MacLab, Castle Hill, Australia), sampled at 10 kHz and recorded in real time and stored as a waveform using Chart software. Recordings were subsequently 100 Hz low-pass filtered and displayed as envelopes of slow waves in arbitrary units. A detailed account of the nerve recording technique can be found in earlier studies (17, 18). Since the effects of the agonists were short-lived, i.e., 2-3 seconds, sympathetic nerve recordings were only qualitatively evaluated to observe the relationship between the microinjection- and/or baroreceptor reflex-induced changes in blood pressure and the sympathetic outflow.

### Baroreceptor reflex sensitivity testing

Baroreceptor reflex sensitivity (BRS) was determined as the ratio of the maximum change in heart rate to the maximum change in mean MAP following an i.v. bolus injection of phenylephrine (25 microg/kg in 0.1 ml saline, Sigma, St Louis, MO). This injection produced a rapid 40-60 mmHg increase in the mean blood pressure. An initial BRS test, to serve as control, was made before the microinjections into the NTS. Sympathetic nerve recordings require the administration of gallamine to prevent artifacts originating from the respiratory movements. As gallamine has the potential to impair the baroreceptor reflex responses due to its vagolytic action, SND recordings were omitted in experiments that include the determination of BRS.

### Sinoaortic denervation

Sinoaortic denervation was made according to the method described by Krieger (19). Briefly, the sympathetic chain, carotid sinus and vagus nerves were isolated, dissected, and then sectioned bilaterally at the level of carotid bifurcation. To verify the success of the denervation two criteria were considered: first, the cardiac-related rhythm in SND was lost; and, second, the reflex inhibition in SND during the phenylephrine-induced activation of baroreceptor reflex (see above) was eliminated.

### Brain stem microinjections

**Figure 1:**
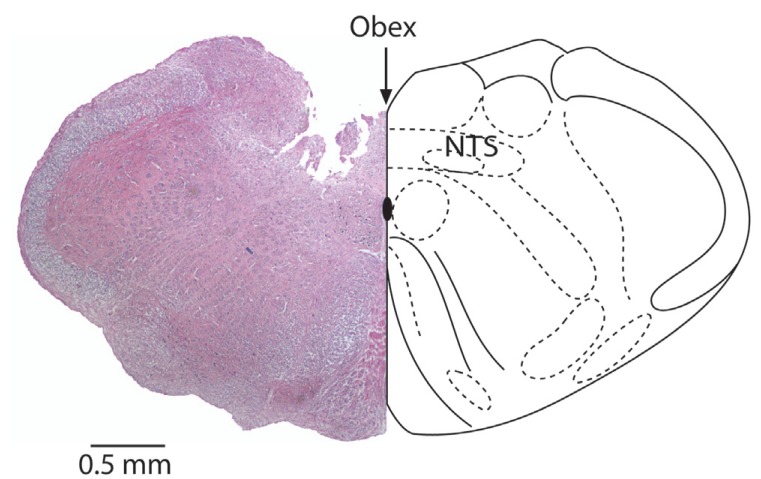
Coronary section of the medulla oblogata at the level of obex showing the microinjection site in the NTS. Calibration bar is 0.5 mm.

Rats were placed in a stereotaxic frame (David Kopf Instruments, Tujunga, CA) with the incisive bar located 10 mm below the interaural line and the dorsal surface of the medulla was exposed surgically through partial removal of the occipital bone. The target stereotaxic coordinates for the NTS were 0.5 mm lateral to the obex and 0.5 mm deep from the brain stem surface. Micropipettes were positioned perpendicular to the horizontal plane by visual guidance. CB receptor agonists WIN 55212-2 (100 mM) and CP 55940 (100 mM) or vehicle (DMSO) microinjections were made in a single-blind fashion in a volume of 0.1 microl using a Hamilton syringe mounted on a custom made micro drive connected to the glass micropipette (1 microm tip diameter) through a polyethylene tubing. One microinjection took about 15 sec to complete. In a separate series of experiments, CB1 receptor antagonist AM 281 (70 mM) or vehicle (DMSO) microinjections were made bilaterally 10 minutes before the agonist microinjections. Bilateral microinjections were made for BRS testing. Only one set of microinjections, i.e. one antagonist followed by one agonist or agonist alone, was made in each animal to avoid manipulation trauma. Each microinjection was individually quantified and data were pooled for subsequent statistical analysis. Since the NTS is a relatively superficial structure, the positioning of the micropipette was performed by visual guidance and, the injection site was measured by naked eye relative to calamus scriptorius (obex) during the procedure. At the end of each experiment, brain stems were removed and histological examination was made in a number of experiments (Figure 1).

### Statistical analysis

Data Analysis of the changes in BRS after microinjections was made with Student’s paired t-test. In experiments in which drug effects were compared, first, a global analysis using ANOVA for repeated measures was performed followed by comparisons between any two groups which were made using Student’s paired t-test. P values less than 0.05 were considered statistically significant. All data in the text and figures were expressed as mean ± SEM.

## RESULTS

### Activation of cannabinoid receptors in the NTS produces baroreceptor reflex-like responses

Microinjections were done blind to the drug labels and they were revealed only after the termination of each experiment set. Histological assessment of the microinjection sites showed that the micropipette tracts were confined within the commissural part of the NTS at the level of obex (Fig. 1).

**Figure 2:**
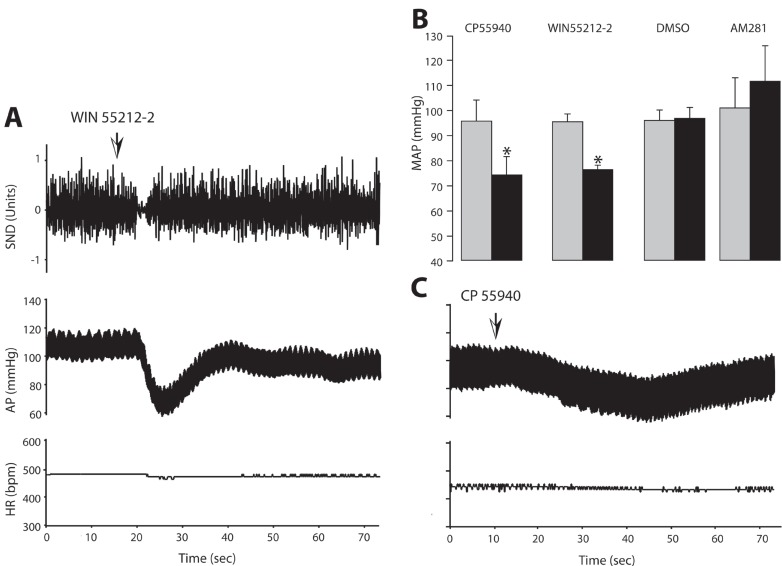
The effects of unilateral microinjection of WIN 55212-2 (Panel A) and CP 55940 (Panel C) on blood pressure, heart rate and splanchnic sympathetic nerve discharge. Arterial pressure (AP) and heart rate (HR) are expressed in mmHg and in beats per min (bpm), respectively. Sympathetic nerve discharge (SND) is expressed in arbitrary units. Panel B: Bar graphs showing the effects of cannabinoid receptor ligands on arterial pressure when microinjected into the NTS. Each bar represents the mean value from 5 experiments ± SEM. ^*^Denotes statistically significant from the control (pre-microinjection) value (*p*<0.05).

In preliminary experiments, NTS microinjections of WIN 55212-2, (3 nmol/0.1 microl) did not produce any change in arterial pressure and SND. Therefore, all microinjections were made using 10 nmol/0.1 microl concentration. WIN 55212-2 microinjection induced an immediate decrease in the arterial pressure which returned to the initial levels in about one minute with a short-lasting inhibition in SND prior to the onset of the blood pressure effects. An example is illustrated in Fig. 2A. This transient decrease in the arterial pressure was statistically significant and the results obtained from these experiments were represented in Fig. 2B. Microinjections of the vehicle DMSO, failed to induce significant changes in the measured parameters (Fig. 2B). In a different set of experiments, microinjections of the other CB receptor agonist CP 55940 (10 nmol/0.1 microl) produced similar hypotensive responses that were also statistically significant (Fig. 2C). Neither WIN 55212-2 nor CP 55940 produced any significant change in the heart rate in experiments in which SND recordings were omitted (data not shown).

**Figure 3:**
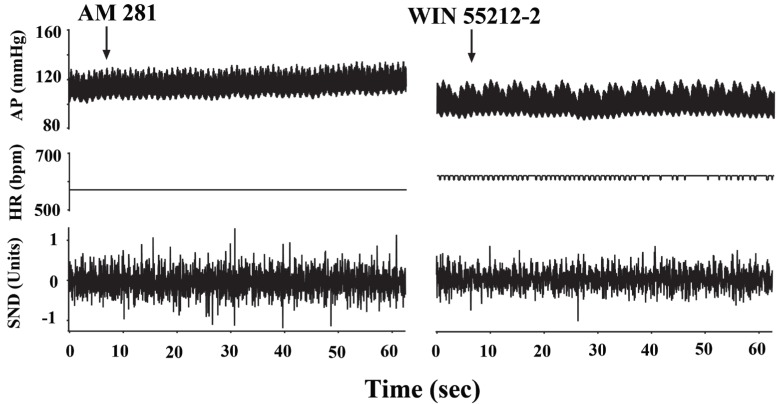
Bilateral microinjection of the cannabinoid receptor antagonist AM 281 did not cause any change in the blood pressure and heart rate but antagonized the actions of WIN 55212-2. Arterial pressure (AP) and heart rate (HR) are expressed in mmHg and in beats per min (bpm), respectively. Sympathetic nerve discharge (SND) is expressed in arbitrary units.

In five rats, 10 minutes before the WIN 55212-2 microinjections, AM 281 (7 nmol/0.1microl) was microinjected into the NTS bilaterally. Following the AM 281 microinjection, arterial pressure showed a steady, albeit not significant increase which returned to the baseline levels within 10 minutes (100.8 ± 12 versus 108.1 ± 12.8mmHg, n=5, *p*>0.05) but prior AM 281 microinjection attenuated the hypotensive responses to WIN 55212-2 (from 95.7 ± 7.4 mmHg to 90.2 ± 6.2 mmHg, n=5, *p*>0.05). An example is given in Fig. 3. In the control group, DMSO microinjections prior to WIN 55212-2 did not produce any significant changes in the hypotensive responses elicited by WIN 55212-2 (108.2 ± 12.9 mmHg to 87.0 ± 14.5 mmHg, n=5, *p*<0.05).

### Bilateral microinjections of cannabinoid receptor antagonist AM 281 into the NTS did not have an effect on the baroreceptor reflex

As cannabinoid receptor agonists elicited baroreflex-like responses, we evaluated their effects on baroreflex responses induced by an abrupt increase in the MAP. Baroreceptor reflex sensitivity (BRS) was expressed as the ratio of the heart rate change over the change in the MAP. The mean BRS, before WIN 55212-2 microinjections, was 1.45 ± 0.36 bpm/mmHg; after AM 281 microinjections, this value was found to be 1.52 ± 0.25 bpm/mmHg. In CP55940 experiments, these values were 1.52 ± 0.25 and 1.73 ± 0.41 bpm/mmHg, before and after AM 281 microinjections, respectively. There was no statistically significant difference between control and post microinjection values as well as between agonist and antagonist microinjections (*p*>0.05, n=5 for each group).

### Non-NMDA receptor antagonist DNQX blocked cannabinoid induced baroreflex like hypotensive responses

**Figure 4:**
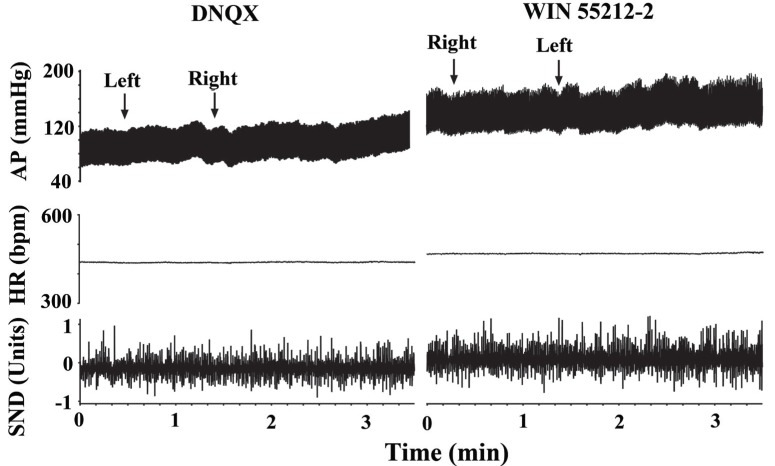
Bilateral microinjection of the non-NMDA receptor antagonist DNQX antagonized the actions of WIN 55212-2. Arterial pressure (AP) and heart rate (HR) are expressed in mmHg and in beats per min (bpm), respectively. Sympathetic nerve discharge (SND) is expressed in arbitrary units.

In five rats, bilateral microinjections of a non-NMDA receptor antagonist DNQX (0.4 nmol/0.1 microl) produced a steady increase of 17.5 ± 5.8 mmHg in the arterial pressure that reached a plateau within approx. 5 min. As illustrated in Fig. 4, bilateral microinjections of WIN 55212-2 (10 nmol/0.1 microl) into the same coordinates 10 min later, failed to elicit the hypotensive responses (92.1 ± 7.9 and 89.2 ± 8.3 mmHg before and after WIN 55212-2, respectively).

### Sinoaortic Denervation attenuated the responses to cannabinoid agonist microinjections

**Figure 5:**
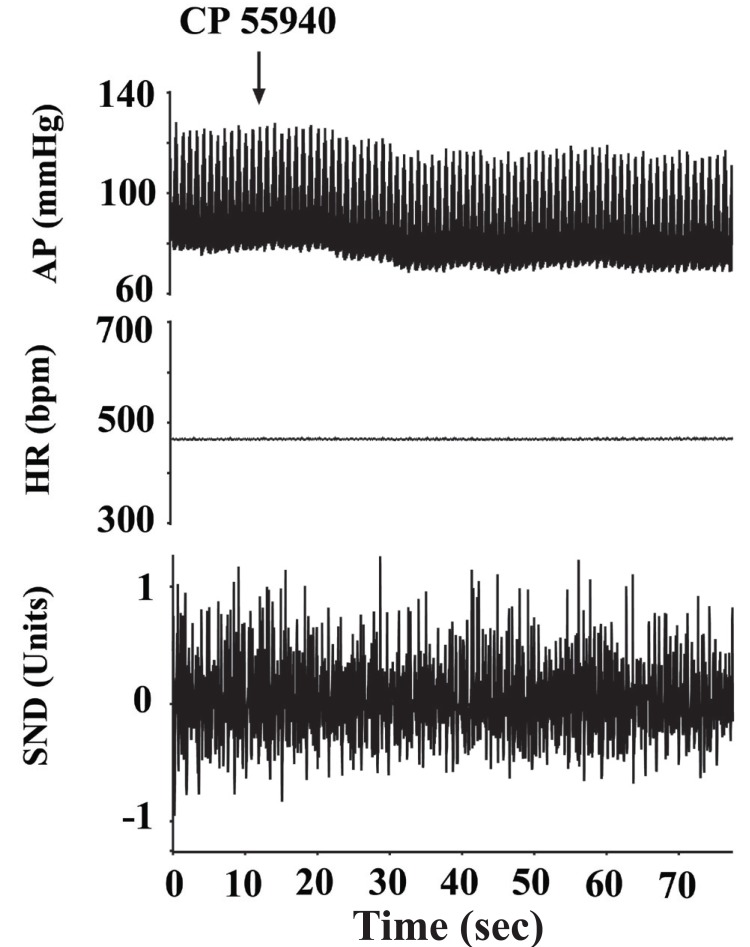
Sinoaortic denervation attenuates the actions of cannabinoid agonist CP 55940. Arterial pressure (AP) and heart rate (HR) are expressed in mmHg and in beats per min (bpm), respectively. Sympathetic nerve discharge (SND) is expressed in arbitrary units.

In seven rats, which satisfied the criteria for sinoaortic denervation, the average baroreflex sensitivity was found to be 0.75 ± 0.24. This value was significantly different from that of intact animals (see above). In sinoaortic-denervated rats, the hypotensive responses to intra NTS microinjection of either WIN 55121-2 or CP 55940 were also significantly attenuated. Since both CP 55940 and WIN 55212-2 produced similar effects in intact animals, data were pooled to express blood pressure changes in sinoaortic-denervated animals. The decrease in MAP following the microinjections of cannabinoid agonists was 8.38 ± 1.58 mmHg. This decrease was not able to produce a statistically significant change compared to pre-injection values (*p*>0.05, n=7). An example is given in Fig. 5.

## DISCUSSION

Our results show that the activation of cannabinoid receptors in the NTS produces baroreflex-like decreases in the arterial pressure. Three major observations support this view: first, the microinjection of two structurally different CB receptor agonists, WIN 55212-2 and CP 55940, but not the vehicle DMSO elicited transient hypotensive responses of similar character; second, the hypotensive responses followed a brief sympathoinhibition and third, these effects were prevented by the pretreatment with CB1 receptor antagonist AM 281. However, neither of these drugs caused any significant change in the heart rate and the baroreflex sensitivity measured by bolus injections of phenylephrine.

The results of the present study are in contradiction with the study by Niederhoffer *et al*., in that the microinjection of cannabinoids into the NTS did not cause any changes in the arterial pressure, while injection into the rostral ventrolateral medulla (rostral VLM) elicited a small sympathoinhibition (15). These authors concluded that NTS is not included among the sites that contribute to the cardiovascular actions mediated by the activation of central CB receptors. Although the agonist concentrations used in these two studies were similar, methodological differences may account for these discrepancies. First, injection methods, i.e., micromanipulator angles, in the commissural NTS were different; second, the volume injected in the present study was twice as much and third, the time base for monitoring the agonist effects differed. In our study, injections were made in 10 to 15 seconds and the response was almost immediate. Whereas, in the aforementioned study, injections were made in 30 seconds and the effects were observed only after 1.5 min. Conflicting results were obtained with CB agonists injected locally into brain stem structures. For example, unlike Niederhoffer *et al*, Padley *et al*. found that the microinjection of cannabinoids into the rostral VLM elicited sympathoexcitation and an increase in the arterial pressure (16). Distribution pattern of CB receptors in the brain stem, particularly in the NTS, may provide an explanation for the transient hypotensive response reported in this study. The density of CB receptors was found to be moderate in the NTS using autoradiographic techniques (19). On the other hand, using immunochemistry, although CB receptors in the NTS were hardly visible at low magnification, “a fine meshwork of thin fibers” was reported at high magnification (20). It could be that the activation of sparsely distributed receptors in a relatively extended structure, like NTS, may be circumstantial in nature and subtle methodological differences may produce different response patterns or fail to produce any response at all.

We think that the actions of the CB agonists in the NTS were mediated through the activation of the baroreceptor reflex. The nucleus of the tractus solitarius is the site where the second order neurons are located which convey the afferent information coming from the peripheral baroreceptors. The reflex hypotension and bradycardia in response to the abrupt rise in arterial pressure is due to the simultaneous activation and inhibition of vagal and sympathetic fibers, respectively.

When baroreceptors are activated by an abrupt increase in blood pressure, NTS activates the CVLM, which in turn inhibits the rostral VLM, the primary site for the projection neurons sending axons to the sympathetic preganglionic neurons located in the intermediolateral column of the spinal cord. Consequently, a decrease is observed in the blood pressure (21).

The fact that there was a short but clear shut off of SND prior to the hypotensive responses following the CB receptor agonist microinjections, suggests that these effects are due to the activation of baroreceptor reflex. We do not anticipate a spread of the drug to the caudal VLM since the two structures are located sufficiently apart to exclude any interference. The injection sites in the current study are close to the dorsal motor nucleus of the vagus. Although a spread to that structure could not be ruled out, the fact that we did not see a bradycardia fails to support a direct vagal activation.

After sinoaortic denervation, cannabinoid receptor agonist microinjections failed to produce baroreceptor activation-like responses, i.e., hypotension and sympathetic inhibition. A possible explanation could be the elimination of tonic glutamatergic input coming from peripheral afferents to the NTS. If this is the case, it is possible that the cannabinoid system acts as to facilitate the glutamate release from afferent fibers in intact animals. However, no direct evidence was obtained to support this hypothesis in this study; on the contrary, it has been shown that cannabinoids act rather to block presynaptic neurotransmitter release in many brain regions (22, 23). In this regard, local GABAergic interneurons seem to be a good candidate for the site of action of cannabinoids in NTS. As explained above, the present study favors cannabinoid receptor activation and there is previously reported evidence that the cannabinoids act as to inhibit synaptic transmission in the NTS (24). However, it is possible that some of the effects of cannabinoids in the NTS could be mediated thorough a yet unknown receptor (25). Further studies are needed to explore these possibilities.

According to our study, an intact baroreflex arc is required to elicit CB receptor-mediated hypotensive responses. Two observations support this hypothesis. First, the responses to WIN 55212-2 were attenuated in sinoaortic-denervated rats; second, the microinjection of non-NMDA receptor antagonist DNQX into the NTS prevented the responses to CB receptor activation. Adding the fact that BRS was unaffected after the agonist and/or antagonist microinjections, we conclude that CB receptors play a modulatory role in baroreceptor reflex responses. In a recent study by Brozoski *et al*., it was shown that the microinjection of an endocannabinoid uptake inhibitor AM 404 into the NTS prolonged the baroreflex inhibition of the SND (26). Together with their previous studies, these researchers concluded that endocannabinoids might play a modulatory role in rapid hypertensive episodes such as seen in labile hypertension (14, 26, 27). Our results are in line with those studies in that an acute perturbation with agonists may produce a baroreflex-like response without affecting the overall baroreflex sensitivity. We therefore suggest that the CB receptors are not involved in the tonic control of the baroreceptor reflex.

Many substances, among them putative neurotransmitters and neuromodulators, have been demonstrated to affect baroreceptor reflex responses in the NTS. Although glutamate is still considered the major neurotransmitter at the first synapse (21), others, including serotonin (12, 13), acetylcholine (28), noradrenaline (29) and substance P (30) have also been shown to modulate the baroreflex in the NTS. Baroreflex control serves as the fastest reacting mechanism to the phasic changes in the arterial pressure and as such, is subject to several physiological as well as emotional influences and other homeostatic inputs. In this regard, it should be emphasized that the modulatory influences may act in a differential manner and it is difficult to assess their contribution under experimental conditions in anesthetized animals. Subtle changes in modulatory influences may have a major role in conditions such as the recovery after exercise (31), the onset of labile hypertension or in chronic dysfunctional stress syndrome (32). Moreover there is evidence that endocannabinoids maybe involved in the pathology of hypotension under conditions like hemorrhage, sepsis, cirrhosis, and myocardial infarction suggesting that they may play a greater role in human and animal pathophysiology than initially anticipated (33). Studies aiming to detect modulatory actions may provide an insight to the mechanisms underlying such conditions.

It is concluded from the present study that cannabinoid receptors in the NTS are not involved in the tonic regulation of the arterial pressure but may have a modulatory role in the baroreceptor reflex integration.
